# Adult mortality and nutrition in rural Senegal: evidence of an epidemiologic transition

**DOI:** 10.1080/16549716.2025.2547493

**Published:** 2025-09-12

**Authors:** Lucie Vanhoutte, Samuel Pavard, Emmanuel Cohen, Laurence Fleury, Géraldine Duthé

**Affiliations:** a UMR7206 Eco-Anthropologie, Muséum National d’Histoire Naturelle, CNRS, Université Paris Cité, Paris, France; b UR15 DEMOSUD, Institut National d’Études Démographiques (INED), Aubervilliers, France; c CNRS, Montpellier, France; d Aix Marseille Univ, IRD, UMR 151 LPED, Marseille, France

**Keywords:** Nutrition transition, verbal autopsy, Health and Demographic Surveillance System (HDSS), non-communicable diseases (NCDs), health transition, Niakhar, Mlomp, Bandafassi, sub-Saharan Africa, global health

## Abstract

**Background:**

Global mortality transitions are driven by the epidemiologic transition, resulting in a rise in non-communicable diseases (NCDs), which are partly shaped by the nutrition transition and associated chronic conditions. In low- and middle-income countries, these shifts are often viewed as primarily urban phenomena. Rural populations may therefore be overlooked in efforts to prevent and manage NCDs, despite facing critical public health challenges.

**Objective:**

This study examines changing patterns of adult mortality and causes of death in rural Senegal to illustrate ongoing mortality, epidemiologic, and nutrition transitions.

**Methods:**

Using data from three rural sites in the Senegalese Health and Demographic Surveillance System, we analysed adult mortality from 1985 to 2020. We calculated all-cause and cause-specific mortality rates among individuals aged 15 to 70 years, based on causes of death determined through verbal autopsy.

**Results:**

Mortality declined across all age groups. Deaths from communicable diseases, maternal conditions, and undernutrition decreased substantially. NCDs have surpassed communicable diseases as the leading cause of death. Causes of death associated with undernutrition have declined, while diet-related NCDs have increased.

**Conclusions:**

Adult mortality is declining in the three rural Senegalese sites studied, due to a decline in epidemics. However, NCDs now pose a major rural health threat, consistent with epidemiologic transition theory. The reversal between mortality patterns associated with undernutrition and diet-related NCDs may signal an ongoing nutrition transition. Strong health systems are crucial for both preventing and treating NCDs, and robust health information systems are needed to support deeper analysis of this issue.

## Paper Context

● **Main findings**: Premature adult mortality (15–70) in three rural areas of Senegal decreased between 1985 and 2020, primarily due to declines in communicable diseases and undernutrition. However, non-communicable diseases, including those related to nutrition, are becoming major health concerns.

● **Added knowledge**: Rural adult populations in low- and middle-income countries (LMICs) are not isolated from the nutrition and epidemiologic transitions which may slow further mortality reductions.

● **Global health impact for policy and action**: Public health policies in poor rural areas must address the growing burden of non-communicable diseases and forms of excess malnutrition.

## Background

Since the eighteenth century, adult mortality has declined worldwide [1]. These mortality transitions are driven by a shift in the leading causes of death from communicable diseases, maternal disorders, and malnutrition (undernutrition) (CMM) to non-communicable diseases (NCDs). Because the onset of the latter is typically delayed, many deaths are postponed, contributing to a steady rise in life expectancy. These epidemiological changes were first documented in western industrialised societies and are conceptualised within the epidemiologic transition theory [2,3]. According to this model, an initial period of high and fluctuating mortality, labelled the ‘age of pestilence and famine’, is followed by a phase of ‘receding epidemics’, during which mortality declines due to advances in the control and prevention of communicable diseases, maternal and neonatal disorders, and undernutrition. As changes in lifestyle and environment unfold, populations enter a phase dominated by ‘degenerative, stress-related and man-made diseases’. Some populations may progress into a fourth phase, characterised by improved management and prevention of NCDs, thereby further extending life expectancy, even as new diseases continue to emerge [2,3]. This framework now serves to describe recent mortality trends in countries undergoing epidemiologic transition.

The epidemiologic transition is closely linked to socio-economic changes, including urbanisation, economic growth, medical advances, and improvements in health systems. Nutrition also plays a central role in these changes. Undernutrition is a major risk factor before the epidemiologic transition, particularly for infants and mothers, as it can be fatal for wasted children and increases the risk of death during childbirth and from communicable diseases such as diarrhoeal infections [4]. In contrast, many adult NCDs (e.g. strokes, heart attacks and diabetes) are associated with suboptimal diets and lifestyles, which become more common in transitional settings.

The role of nutrition in the epidemiologic transition is conceptualised within the ‘nutrition transition’. According to this framework, developed by B. M. Popkin [5], most of human history was shaped by dietary patterns focused on ‘collecting food’ and later, on coping with ‘famine’. More recently, and at varying times and rates, human populations have entered a phase of ‘receding famines’, followed by a phase marked by ‘degenerative diseases’ associated with suboptimal diets and lifestyles. Modern diets tend to include more processed foods, added sugars, fats, and salt while physical activity decreases. This has led to a ‘double burden of malnutrition’ in many places, where undernutrition and overnutrition coexist. This article will use ‘new-malnutrition’ to refer to overnutrition and diet-related NCDs that include obesity, type 2 diabetes, cardiovascular diseases, hypertension, and certain cancers.

During both the epidemiologic and nutrition transitions, the burden of disease and mortality shifts from infants, and women of reproductive ages, to older adults. These transitions are often examined through their positive effects on infant mortality, while adult health has received comparatively less attention, particularly in low-resource settings where data on adults are limited [6,7]. This gap is especially pronounced in nutrition research, where child undernutrition -often coupled with nutritional status of mothers- is better documented and sometimes seen as more urgent than malnutrition among the general adult population [8–10].

The epidemiological shift has major implications for health systems. In low- and middle-income countries (LMICs), where access to treatment for chronic diseases remains limited, prevention is critical to reducing premature adult mortality, defined by the World Health Organization (WHO) as death before age 70. However, the conditions to which health systems in LMICs must respond are often poorly described, particularly in rural areas, which are frequently underrepresented in national statistics and health system coverage [11,12]. These areas are commonly perceived as insulated from globalisation-related phenomena due to lifestyles centred on subsistence agriculture and foraging, which require high energy expenditure. This presumed isolation has contributed to limited interest in studying rural health transitions.

Sub-Saharan Africa has the lowest availability of mortality data, largely due to inadequate death registration systems. According to the United Nations Statistics Division (UNSD), as of 2023, 23 of 50 countries in the region had no data on death registration, and 7 others reported registration rates below 50% [13]. In Senegal, the 2013 census found that only 30.8% of deaths were registered nationally, and just 12.4% in rural areas [14]. Although mortality and cause-of-death estimates exist for Senegal and most other countries, thanks to initiatives such as the Global Burden of Disease project (GBD [15]) and the WHO Global Health Observatory [16], these estimates are not data-driven. Rather, they rely on complex and speculative modelling techniques and, as their authors note, should be interpreted with caution. Meanwhile, growing evidence indicates that urban areas in sub-Saharan Africa are exposed to new health and mortality risks [17–19]. Emerging research on the nutrition transition also suggests that rural areas may face rising exposure to NCDs, which were previously concentrated in urban settings that had been driving the epidemiologic transition. However, the extent of this shift in sub-Saharan Africa remains unclear [20,21].

The lack of reliable data on recent adult mortality trends in rural areas of low-income sub-Saharan countries creates a pressing need for updated, context-specific information on how mortality, epidemiologic, and nutrition transitions are shaping premature adult mortality in these settings.

Senegal is a lower-middle-income country in sub-Saharan Africa, which remains predominantly rural, though it is becoming increasingly urbanised and integrated into global economic systems [22,23]. Between 2009 and 2019, food imports, particularly rice and wheat, increased at an annual rate of 4.3%, outpacing annual population growth, which was 2.7% during the same period references [24]. This trend may reflect gradual shifts in Senegalese diets, further suggested by the current composition of the national diet [25].

Despite signs of economic and social change, its impact on health over time remains difficult to assess. Many studies on the epidemiologic and nutrition transitions in sub-Saharan Africa focus on declining infant mortality while adult mortality, especially over the long term, remains under-researched due to data limitations [7,26–28]. In Senegal, high-quality mortality data are available for three rural areas -Bandafassi, Mlomp and Niakhar- between 1985 and 2020, through Health and Demographic Surveillance Systems (HDSS) [29–31]. HDSS data have supported several studies on mortality and/or nutrition [8,32–36] but none of them studied in detail cause specific adult mortality trends in relation with the nutrition transition.

This study examines trends in adult mortality to assess whether a transition is evident among adults in rural Senegalese sites. We further explore how the epidemiologic transition, particularly its nutritional dimension, is reflected in premature adult mortality (ages 15 to 70), using pooled data from the three sites for the period 1985 to 2017. Our analysis is structured around three nested models: the mortality transition, the epidemiologic transition, and the nutrition transition. We hypothesise that all three transitions are underway in rural Senegal and are observable through changes in mortality patterns.

For the mortality transition, we expect a decline in mortality across all age groups. For the epidemiologic transition, we anticipate a shift in the leading causes of death from CMM to NCDs. For the nutrition transition, we expect a decline in mortality from causes linked to undernutrition, and a rise in mortality from causes associated with suboptimal diets and lifestyles.

To test these hypotheses, we first examine trends in all-cause mortality over the period, then compare NCD and CMM mortality, and finally analyse the share and rates of nutrition-related causes of death among adults.

## Methods

### Data

HDSSs are population-based information systems used in regions where national statistics are incomplete. They involve prospective surveys, and exhaustive tracking of key demographic events, namely, births, deaths, unions, and migrations. In Senegal, causes of death within HDSS areas are determined using the verbal autopsy method whereby, for each death, a relative of the deceased is interviewed about symptoms, medical history, and circumstances preceding death [37]. Physicians then review the responses to assign the most probable biomedical cause of death.

The Senegalese HDSS is operated by *Observatoire Population, Santé, Environnement* (OPSE), a consortium of French and Senegalese institutions. This study examines three OPSE sites: Bandafassi in the Kedougou region of south-eastern Senegal (monitored since 1980), Mlomp in the Ziguinchor region of southern Senegal (monitored since 1985), and Niakhar in the Fatick region (monitored since 1983) [29–31]. Mlomp is 50 km from the regional capital, Ziguinchor, Niakhar is 135 km from Dakar; and Bandafassi is 250 km from Tambacounda, the nearest large city. Agriculture dominates these areas: Bandafassi grows cereals, peanuts, and cotton; Mlomp cultivates rice; Niakhar produces peanuts and millet. Cattle breeding occurs in Bandafassi and Niakhar. As rural areas, all three sites face challenges in accessing healthcare, often due to geographic or economic barriers, particularly in Bandafassi, where the population is dispersed across isolated villages. In contrast, Niakhar’s villages are better connected by road infrastructure, and Mlomp benefits from a compact population, a local dispensary, and easier road access to additional health facilities.

This study utilised three types of information from the OPSE databases:

- Event tables recording each individual’s entry and exit events (census, immigration/emigration, birth/death, last observation).
- Personal information tables, including sex and date of birth.
- Death and cause-of-death tables, providing date of death and assigned cause of death, when available, coded per the ninth revision of the International Classification of Diseases (ICD-9).

To standardise death rates, we applied the sub-Saharan Africa standard population developed by the INDEPTH Network [38].

### All-cause mortality

#### Population, age groups and periods

The observation period spans from 1 January 1985 to 31 December 2020, representing the longest consistent timeframe across all three sites. We focused on premature adult mortality, selecting individuals aged 15 to 70 who were under observation during this period. The population was stratified by sex, year and age group. The dataset is further described in Table 1.

**Table 1.** Description of the data used for all-cause mortality analysis, Senegal HDSS, 1985–2020, population aged 15–70.

	Bandafassi	Mlomp	Niakhar	Total
Individuals on 01/01/1985	4,209	3,198	10,234	17,641
Individuals on 01/01/2020	7,380	4,445	24,780	36,605
Individuals observed, 1985–2020	14,736	14,620	51,446	80,802
*Proportion of women (%)*	*51.9*	*48.1*	*53.9*	*52.5*
Person-years, 1985–2020	207,163	172,156	636,663	1,015,983
Deaths 1985–2020	1,540	899	3,642	6,081

Source: OPSE data (authors’ calculations).

#### Analyses

All analyses were conducted using R (version 4.1.2 ‘Bird Hippie’) [39]. Standardised death rates were calculated for each site and sex, for 3-year periods at both the beginning and end of the study period. Smoothed death rates were computed for each site, sex, year and age using the R function Mort2Smooth (package MortalitySmooth, version 2.3.4) [40], with default settings (i.e. 17 splines for age and 7 splines for years). Smoothing was applied to reduce variability caused by the small size of each year-age group. This method assumes no large outliers in the data. To prevent distortion from a singular event, we censored the numerous deaths registered at the Mlomp site following the Joola ferry sinking, a maritime disaster which resulted in 55 deaths within our sample on 26 September 2002. These deaths were deemed unrepresentative of mortality trends and causes in the region and were therefore censored (i.e., individuals are considered lost to follow-up, rather than deceased, from 26 September 2002 onward).

### Cause-specific mortality

#### Population, age groups and periods

Given the small number of deaths per year, site, sex, and age group, along with the further subdivision into cause-specific categories (see below), we reduced the granularity of these variables by increasing the sample size. Since mortality trends directions by age and over time were similar across the three rural sites, and there is no evidence of site-specific cause-of-death patterns, we pooled the sites. We also grouped ages into two categories (15–49 and 50–69) and years into twelve 3-year periods, from 1985–1987 to 2018–2020.

We then accounted for incomplete cause-of-death data, due to the presence of both ill-defined and missing causes (see Figure S1 in the Supplemental material for details on the distribution of incomplete data).

First, ill-defined causes refer to deaths for which the verbal autopsy did not yield enough information for physicians to assign a specific cause, in the absence of a formal medical examination (with the exception of Niakhar until 1997, where an ICD adaptation was used, resulting in unknown causes of death (N/A, see below) being classified as ill-defined). We did not treat these as missing data, since the verbal autopsy process was completed and yielded partial information. As this partial data can sometimes allow classification into broad categories, we retained ill-defined causes in the dataset without modification. The limitations introduced by ill-defined causes depend on how sensitive the verbal autopsy method is to the different aggregated cause-of-death categories we consider. However, to our knowledge, no consensus exists on whether verbal autopsies are more sensitive to deaths from communicable or non-communicable diseases. This uncertainty is compounded by the fact that diagnostic sensitivity varies with the epidemiological context [41]. Accordingly, we assessed the effect that overrepresentation in ill-defined causes of any of our interest groups might have on interpretation.

On the other hand, some causes of death were strictly unavailable (marked as ‘Not Available’, or N/A), due to a range of factors. Their proportion varied by site, period, and age group (see Figure S1). In some cases, specific groups were excluded from the verbal autopsy protocol. For instance, in Niakhar between 1998 and 2004, causes of death were investigated only for individuals under the age of 55. In other cases, no relative was available to perform the verbal autopsy, or the information was collected but not recorded in the database at the time of this study. Additionally, the COVID-19 pandemic disrupted the verbal autopsy process in 2020 and 2021, when data were being retrieved.

Overall, 24% of causes of death were N/As, with the proportion ranging from 0.8% to 100% across different time periods, age groups, and sites. We selected a subset of groups in which the share of N/As was less than 25%. This threshold was chosen because 98% of the groups had either less than 20% or more than 30% of N/As. We decided not to impute the missing causes because neither the literature nor the data distribution supports assumptions about the missing data structure. This subsetting led to the exclusion of certain sites from the pooled sample in specific periods (see Figure S2 in Supplemental material for the distribution of the study population by age, period, sex, and site), and to the exclusion of the 2018–2020 period altogether. In the final sample, the proportion of N/As was reduced to 6.9%. The total number of person-years observed in this restricted population is 778,118 (see Table S1 in Supplemental material).

#### Causes of death classification

To test the epidemiologic transition hypothesis, we group all causes of death into five broad categories (1): communicable diseases, maternal disorders and undernutrition (‘CMM’) (2); non-communicable diseases (‘NCDs’) (3); injuries and external causes (‘injuries’) (4); ill-defined causes (‘ill-defined’); and (5) unavailable cause of death (‘N/A’). The CMM, NCDs, and injuries categories encompass all deaths with a determined cause. This classification is based on the system used by both the GBD and WHO, which reflects the structure of causes of death theorised in the epidemiologic transition model. The only difference from the standard classification used by the GBD and WHO is the exclusion of ‘neonatal disorders’ from the CMM category, as these are not relevant to our study of adult mortality.

To explore the nutrition transition, we excluded non-nutritional causes of death, and focused on those potentially influenced by poor nutritional conditions: either undernutrition, typically prevalent before the transition (‘undernutrition-related causes’) or suboptimal diets and lifestyles more prevalent during the transition (‘new-malnutrition-related causes’). Nutritional causes are rarely identified as primary causes of death through the verbal autopsy method. To select relevant causes, we relied on the GBD framework, which links causes of death with risk factors. We used correspondence tables from https://vizhub.healthdata.org/gbd-results/to identify relevant risk – cause associations, drawing on the GBD risks hierarchy, causes of death hierarchy, and their interrelations. We followed the selection undertaken by the GBD Risk Factors Collaborators, who identify risk – cause links supported by ‘convincing or probable evidence of causation’ in the scientific literature [77,78]. We selected causes of death associated by the GBD with the following nutrition-related risk factors: from which we selected those nutrition-related. ‘Child and maternal malnutrition’ was used as the risk associated with undernutrition. As a result, undernutrition-related deaths included in our analysis were those coded in the ICD-9 as caused by ‘nutritional deficiencies’, by certain communicable diseases (especially intestinal infections and some parasitic diseases), or by ‘complications of pregnancy, childbirth, and the puerperium’, conditions often exasperated by undernutrition. ‘Low physical activity’, ‘dietary risk’, ‘High fasting plasma glucose’, ‘High LDL cholesterol’, ‘High systolic blood pressure’, ‘High body-mass index’, were used as risks associated with new-malnutrition. As a result, new-malnutrition-related causes included select neoplasms (particularly those of the colon and rectum), ‘diseases of the circulatory system’, and ‘nutritional and metabolic diseases’. They also include tuberculosis and dementia (in the GBD classification, Alzheimer diseases and other dementias are associated with ‘High fasting plasma glucose’ and ‘High body-mass index’; tuberculosis is associated with ‘High fasting plasma glucose’, and not associated with ‘child and maternal malnutrition’).

#### Analyses

We measured the proportion of each cause-of-death category over time. We then calculated cause-specific, age-standardised death rates.

## Results

### An evident mortality transition in all-causes mortality trends

In the pooled study population, mortality declined by approximately half between 1985–1987 and 2018–2020. This decline was more pronounced in Bandafassi and Niakhar than in Mlomp, where mortality was already lower in the late 1980s. By 2018–2020, mortality levels were similar across all three sites (see Table 2).

**Table 2.** Standardised death rates (Senegal HDSS), 1985–2020, population aged 15–70.

Site	1985–1987	2018–2020	Difference (%)
All sites	0.0082	0.0037	−54
Bandafassi	0.0105	0.0037	−65
Mlomp	0.0045	0.0037	−18
Niakhar	0.0095	0.0039	−59

Source: OPSE data (authors’ calculations).

**Figure 1.**
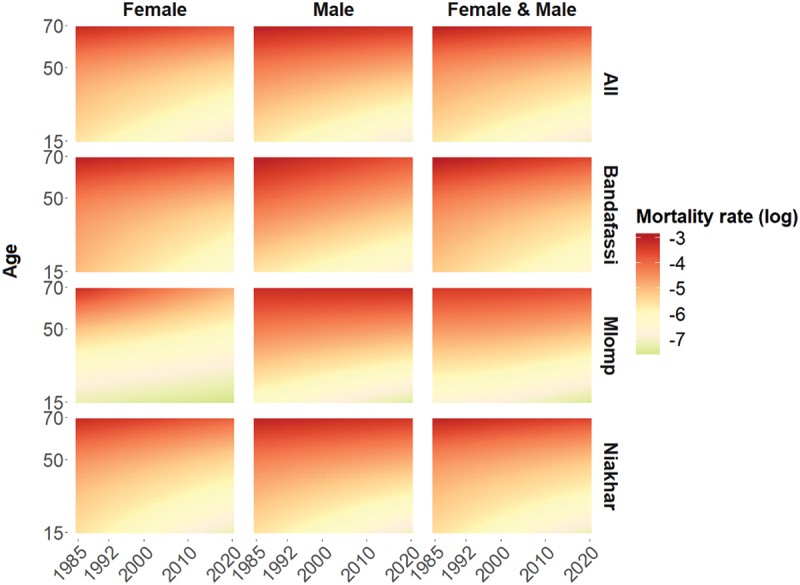
Death rates in Senegal HDSS, 1985−2020, population aged 15–70. Source: OPSE data (authors’ calculations)

Figure 1 is a heatmap showing smoothed mortality rates for all ages and years. It reveals a decline in mortality for all ages across all sites between 1985 and 2020, especially since 2005–2011, primarily due to the widespread use of artemisinin-based combination therapy to reduce malaria mortality [42,43].

However, this progress has been more pronounced among younger ages. Between 1985 and 2020, smoothed mortality rates for individuals aged 15 years decreased by 78%, 73%, and 53% in Niakhar, Bandafassi, and Mlomp, respectively, with an overall decline of 76% across all sites. Mortality reductions were less pronounced among older age groups, with a 58% reduction for adults aged 50 and a 51% reduction for adults aged 69. High mortality rates among adults shifted to older age groups, though it is worth noting that mortality among men aged around 60 in Mlomp has not decreased since 1985.

Overall, mortality rates for men are consistently higher than for women across the study period. Elevated mortality rates among young women suggest unmet reproductive health needs, particularly in Bandafassi, where maternal mortality appears to have declined only in the past decade. In Mlomp, where health care access for women of reproductive age is greater [30], the gender gap in mortality is widest, favouring women.

The sites have few specific characteristics. Mlomp had lower mortality rates than the other two sites at the beginning of the study period, so improvements were more modest. Bandafassi shows higher mortality rates among young women, which may indicate elevated maternal mortality. However, a clear trend towards an overall reduction in mortality is evident at all three sites.

The reduction in premature mortality across all ages, with a more pronounced decline among young women, aligns with the mortality transition described in the epidemiologic transition. Maternal mortality serves as a key indicator of the decline in epidemics and adult undernutrition [44]. The more limited progress for men and older adults raises important questions that can only be addressed through cause-specific analysis.

### Cause-specific mortality reflecting epidemiological transformations

#### Cause-specific analysis suggests a plausible shift from communicable to non-communicable diseases

**Figure 2.**
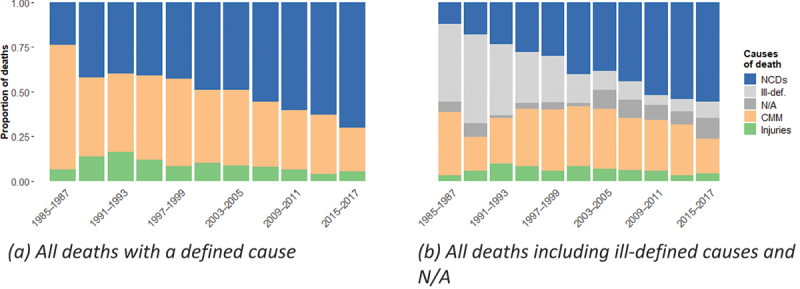
Distribution of deaths by broad cause group and period (Senegal HDSS), 1985–2017, population aged 15–70. Source: OPSE data (authors’ calculations)

Figure 2 presents the relative share of mortality attributed to CMM, NCDs, and injuries.

Figure 2a illustrates an epidemiologic transition. The relative share of mortality due to CMM declines, while NCDs become the leading causes of death.

Figure 2b highlights an element of uncertainty. Although the share of NCDs increases throughout the period, the proportion of deaths attributed to ill-defined or unavailable causes decreases. This pattern suggests that the apparent rise in NCDs may be partly due to improved diagnostic precision which reclassifies previously ill-defined causes of death as NCDs. If all ill-defined and unavailable causes were assumed to be NCDs, the relative share of NCDs compared to CMM would remain stable. However, this assumption would imply that only NCDs are underdiagnosed.

In reality, ill-defined deaths likely include both CMM and NCDs. This is due to (i) diseases in both groups with low sensitivity and specificity in the verbal autopsy methods, (ii) some causes inevitably remaining ill-defined. In 1988–1990 (Figure 2b, second period), a spike in ill-defined and unavailable causes coincides with an unexpectedly low share of CMM, while the proportion of NCDs continues to rise steadily. This suggests that reclassification from ill-defined and N/A causes affects both CMM and NCDs. We conducted a sensitivity analysis that suggested that the share of NCDs in the unknown causes (ill-defined and unavailable) should be superior to 75% for the transition to be blurred, which is a very high and very improbable overrepresentation of NCDs especially at the beginning of the period (which has the greatest impact on the results). Therefore, it is unlikely that no transition from CMM to NCDs occurred. Rather, the extent of the transition is difficult to quantify due to diagnostic uncertainty at the beginning of the study period.

**Figure 3.**
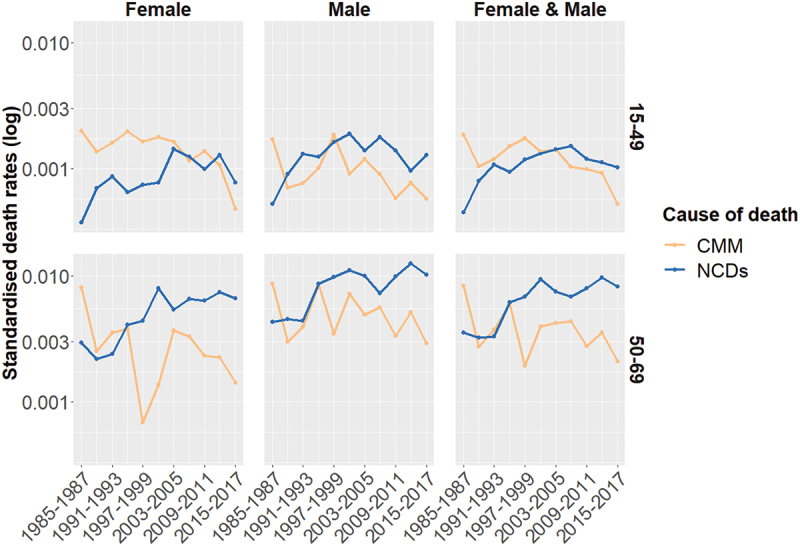
Standardised NCD- and CMM-specific death rates, by period and age groups, Senegal HDSS, 1985–2017, population aged 15–70. Source: OPSE data (our calculation)

This transition may be driven either by shifts in risk factors or demographic changes, such as population ageing, which increases exposure to NCDs. To isolate changes in cause-specific mortality from the effects of age, we present age-standardised cause-specific death rates for CMM and NCDs in Figure 3.

Figure 3 shows that the decline in mortality among adults aged 15–70 is driven by a sharp reduction in CMM. This confirms having already entered the first phase of the epidemiologic transition, namely the decline in CMM mortality. At the same time, the study areas also appear to be experiencing rising rates of NCD-related deaths, independent of the population age structure. NCD-related death rates are particularly increasing among adults aged 50–70 and appear to have surpassed CMM as the leading cause of death during the early 1990s. In 2015–2017, the leading NCDs were cerebrovascular diseases (12.6% of deaths), followed by malignant neoplasms of the digestive organs and peritoneum (4%) and hypertensive disease (4%). Pneumonia/influenza (5%) and tuberculosis (3.5%) were the most frequent communicable causes, ranking second and fifth, respectively, among the top five. In contrast, in 1985–1990, the top three causes of death were all communicable diseases: intestinal infectious diseases (10.5% of all deaths), tuberculosis (4%), and pneumonia/influenza (4%).

While the increase in NCD mortality among adult ages 50–70 may reflect an expected shift from CMM to NCDs, the rise among those aged 15–49 is somewhat unexpected. Although smaller in magnitude than the increase among older adults, it may signal changing behavioural and environmental risk factors, such as pollution, reduced physical activity, or unhealthy consumption patterns. The following section explores the role of nutrition-related risks in this transition.

#### Observable shift in nutrition-related causes

To further explore the transition and assess whether emerging risks are limiting the decline in premature mortality, we focus on the nutritional dimension of the epidemiologic transition, that is, the nutrition transition.

**Figure 4.**
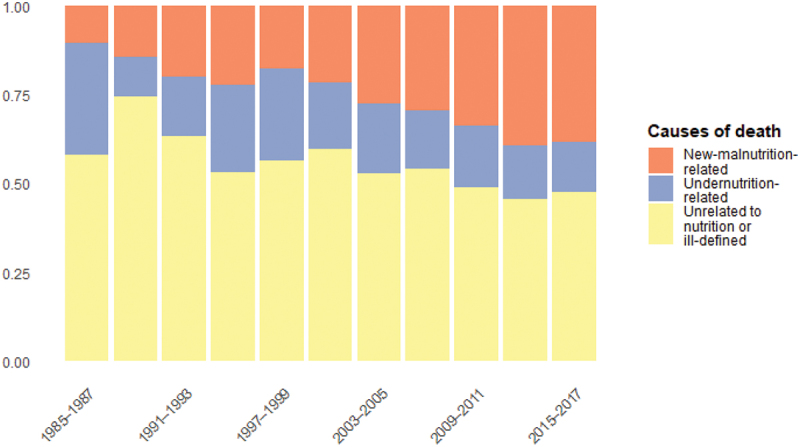
Proportion of defined causes of death potentially attributable to nutrition (Senegal HDSS), 1985–2017, population aged 15−70. Source: OPSE data (authors’ calculations)

Figure 4 shows trends in the proportion of undernutrition-related versus new-malnutrition-related causes of death among all deaths with a defined cause. Over time, the leading nutrition-related causes shift from undernutrition-related (representing 74% of such deaths in 1985–1987) to new-malnutrition-related (representing 69% in 2015–2017).

**Figure 5.**
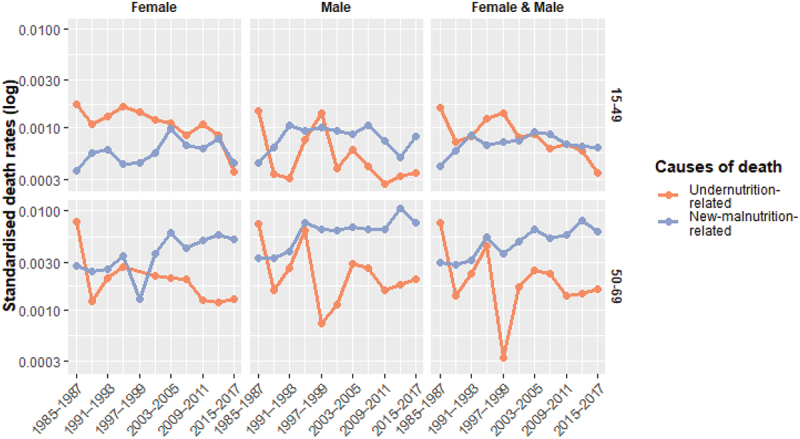
Standardised death rates for nutrition-related causes of death (Senegal HDSS), 1985−2017, population aged 15−70. Source: OPSE data (authors’ calculations)

The shift in nutrition-related causes of death may be partially explained by changes in the populations age structure. However, both younger and older adults age groups are vulnerable to undernutrition, so an ageing population does not necessarily imply a decline in undernutrition. Figure 5, which presents standardised cause-specific mortality rates, shows that undernutrition-related mortality has declined in the study areas, though not in a linear fashion. At the same time, deaths attributable to suboptimal diets and lifestyles have increased among adults aged 50 − 70, and may have also risen, albeit to a lesser extent, among younger and middle-aged adults.

The potential impact of ill-defined deaths warrants consideration here as well (see Figure S3 in Supplemental material). Given the substantial decline in the proportion of ill-defined causes over time, any ill-defined causes that truly belong to the undernutrition category would only accentuate the observed decline. Conversely, the upward trend in new-malnutrition-related causes would be flattened. Moreover, since 40% of defined causes in our database are excluded from this analysis as not nutrition-related, it is reasonable to assume that a similar proportion of ill-defined deaths are likely unrelated to nutrition. These would not be included in Figure 5, even if the exact cause of death were known.

## Discussion

The OPSE dataset stands out among health and demographic longitudinal data collected in rural areas of lower-middle-income countries in sub-Saharan Africa. The recording of vital events such as births and deaths is highly reliable compared to civil registration systems or censuses in Senegal [23,45]. Although data collection methods vary across the three OPSE sites and over time, these differences do not compromise the validity or consistency of the mortality data, including cause-of-death information. As a result, the dataset provides a strong basis for analysing overall mortality trends across the three sites considered collectively [46]. The analyses presented here provide a valid approach to reliably identifying major mortality trends and assessing whether the data support the presence of ongoing epidemiologic and nutrition transitions.

The findings show that the mortality transition is underway in rural Senegal, including among adults. Although our study stops in 2020 (causes of deaths occurred in 2020 and later were not available at the time of the writing of the paper), there is no reason to believe that there was an outbreak of infectious causes since the end of our study period). Covid-19 in particular did not impact mortality in the region. Adult mortality should be studied with the same level of attention as infant mortality, even in remote areas. The decline in CMM-related mortality indicates that these rural areas have entered the epidemiologic transition. Our findings also highlight, in a novel way, that the crossover between CMM and NCDs as leading causes of death is a useful indicator of changing mortality patterns in rural contexts. Questions remain as to whether this crossover results in a net reduction in mortality, or whether NCD-related deaths are partly offsetting these gains. Given the relative uncertainty surrounding some of the data, the central unresolved issue in our analysis concerns not whether NCDs currently affect adult mortality in rural areas, which is clearly the case, but whether their role has been underestimated in the past. What is evident is that the impact of NCDs is becoming more visible as other causes of death decline.

The findings support the conclusion that rural areas in Senegal are experiencing epidemiological shifts similar to those observed in more urbanised settings [12,19,47–51]. Moreover, the results are consistent with the hypothesis that behavioural and environmental changes, particularly those related to nutrition, are key drivers of the observed epidemiological changes. However, while the trends presented here are robust, precisely measuring these changes remains challenging due to the absence of detailed, cause-specific mortality data in Senegal.

Nonetheless, the implications for public health policy are clear. Programs that focus solely on communicable diseases and undernutrition risk overlooking the majority of adults seeking care in rural areas. Addressing this gap requires essential multisectoral, integrated strategies for preventing and managing NCDs in isolated rural areas, such as those recommended by the World Health Organization [52,53]. Yet, despite Senegal’s efforts to improve NCD surveillance and health infrastructure, a persistent shortage of trained health professionals remains a major barrier to progress, especially in remote areas.

This study is among the few to examine longitudinal trends in adult mortality in rural sub-Saharan Africa and, to our knowledge, the only one to focus specifically on nutrition-related causes of death. Although LMICs are taking steps to better assess the prevalence and risk factors of NCDs, health information systems and representative surveys rarely support retrospective analyses or allow for measurement of the impact of changing morbidity on population-level mortality. Previous studies have explored indications of the nutrition transition in West African countries, but they typically focus on proxy morbidity indicators, especially obesity, which may divert attention from other nutrition-related chronic diseases. This focus may also contribute to underestimating the extent of the nutrition transition in rural areas, as the urban – rural differential is often more pronounced for obesity [54]. In contrast, analysing causes of death captures all fatal outcomes potentially linked to nutrition.

Our findings regarding the epidemiologic and nutrition transitions, though qualified, align with ethnographic studies in Senegal, that document transitional changes in diet and health among rural populations [55–58]. These lifestyle changes are consistent with macro-economic shifts at the national level. While Senegal remains heavily reliant on small-scale agriculture, the declining contribution of agriculture to gross domestic product is consistent with trends in urbanisation and tertiarisation, that is, the expansion of the service sector [22,23,59]. Our results also correspond with existing epidemiological studies that suggest an ongoing epidemiologic transition and a complex picture regarding the nutrition transition. Although this phenomenon has been more extensively studied in urban contexts, recent findings indicate similar trends in rural areas, corroborating our own.

A 2019 longitudinal study in a health facility in Dakar found that consultations for NCDs increased by 7% annually between 2005 and 2014, suggesting a rapid epidemiologic transition in urban Senegal [48]. Another study reported a high prevalence of hypertension and obesity in Dakar [60]. In rural Mlomp, a 2008 study highlighted the importance of NCDs in adult mortality, findings echoed in our analysis of all three sites [61].

With regards to nutrition, a 2015 STEPwise survey [62] in Senegal found high rates of diabetes and hypertension among older adults in rural areas, particularly among women [49,63]. A comparative study from the same year revealed that although the nutrition transition was more advanced in urban areas of Senegal, the urban – rural gap was narrowing [51]. Several studies have attempted to assess Senegal’s stage in the nutrition transition. In 2011, Abrahams et al. proposed a cross-sectional score that ranked Senegal among the most advanced West African countries in the transition, though this score was based solely on early life indicators [27]. A more holistic analysis by Cohen et al., using multiple criteria in both rural and urban areas, found that Senegal’s nutrition transition was real but still in its early stages [50]. Similarly, a qualitative study by Rapinski et al. in the rural Ferlo region of Senegal noted the onset of the nutrition transition, but found that it had not reached the stage of ‘man-made, stress-related, and degenerative diseases’, as observed in European and American contexts [56]. A 2022 study on diets and the nutrition transitions in 43 sub-Saharan African countries, including Senegal, emphasised that indicators of transition often coexist with signs of persistent food insecurity [25]. The mixed results in the literature mirror the findings of our study, highlighting an incomplete ‘receding famine’ phase alongside the rising burden of degenerative diseases linked to dietary and lifestyle changes. For a more comprehensive understanding of these transitions, future research might compare our results with similar data collected in urban Senegal [64].

Several limitations of this study should be acknowledged. The three HDSS sites were implemented at different times by separate teams, with some variations in operations over the long study period [31,65,66]. As such, valuable longitudinal depth may be affected by some loss in consistency. To produce a coherent series of causes of death over time while minimising the proportion of missing data, we had to balance population size with site coverage. Notably, for adults aged 50–70 during the period 1997–2002, our dataset includes only cause-of-death data from Mlomp. This imbalance in site representation (see Figure S2 in Supplemental material) partly accounts for the variability seen in Figures 3 and 5. The decision to pool the three sites together entails a loss of specificity. However, this decision was the most reasonable considering, on the one hand, our research question, that is transitions in rural Senegal (as opposed to urban) and not the comparison between several rural settings, and on the other hand, the limitations of the data for each of the three sites (incomplete data in Bandafassi, very small numbers in Mlomp, exclusion of part of the population during some periods in Niakhar). More broadly, this study adopts a balanced approach between the raw description of inherently incomplete data and the adjustments necessary to reveal underlying trends in an interpretable manner. This tension is common when conducting research in low-resource settings, where data limitations are a persistent challenge.

The verbal autopsy method generates data influenced by various factors and must therefore be interpreted with caution [37]. One potential bias is the initially high proportion of ill-defined causes of death, whose distribution across cause categories is difficult to hypothesise and may be biased by issues such as under-diagnosis, a limitation shared with other cause-of-death identification methods. This issue was first analysed by Preston and Nelson in 1974 [67] and has since been further studied, particularly in relation to major communicable diseases. For example, a literature review by M. Anker (1997) found that verbal autopsy sensitivity for communicable diseases in children varies widely by context [68]. The main concern in our study is the potential difference in sensitivity between cause-of-death categories. That is, one group of causes may be appreciably more likely to be classified as ill-defined than another (NCDs vs. CMM, or undernutrition-related vs. new-malnutrition-related causes). To our knowledge, the sensitivity of verbal autopsies for identifying the most common causes of death has not been assessed in the Senegalese OPSE. Chandramohan, Setel, and Quigley cautioned that sensitivity estimates from one setting should not be assumed to apply to another with a different cause-of-death profile. For instance, one validation study found that diarrhoeal diseases (CMM) were more accurately diagnosed than cardiovascular diseases (NCD), while another found the reverse [41]. Due to this high degree of uncertainty, we chose not to impute unavailable or ill-defined causes of death, as doing so would have required assumptions about the structure of the missing data. Further studies are therefore needed to assess differences the difference in verbal autopsy sensitivity between NCDs and CMM in rural Senegal.

Another potential bias in verbal autopsies is reduced specificity for certain cause-of-death categories, which can lead to overestimation of one group relative to others. On this point, Yang et al. noted that ‘[verbal autopsy] is an imprecise tool for detecting leading causes of death among adults, however, much of the misclassification generally occurs within broad cause groups (e.g. CVD, respiratory diseases, and liver diseases)’ [69]. Because our groups are defined more broadly, such within-group misclassification does not affect our results.

Uncertainty regarding verbal autopsy bias does not fundamentally undermine the applicability of the transition models, as these models were also developed using data subject to bias and uncertainty.

Another limitation of our analysis is that the attribution of deaths to risk factors applies only to a subset of cases, and the strength of evidence varies across risk – cause pairs [70,71]. Our nutrition-related classification includes both causes of death closely linked to nutrition and those only partially attributable to nutrition or metabolism. As with any classification, a degree of arbitrariness is unavoidable. For instance, we consider food safety risks to be characteristic of a pre-nutrition-transition context; however, in low-resource urban environments, contamination and intoxication risks may increase [24,72]. Tuberculosis is another example of a cause of death that could reasonably be assigned to more than one category. The Global Burden of Disease classifies it as associated with diabetes, and not associated with maternal and children undernutrition, which informs our classification of tuberculosis as one of the causes associated with the nutrition transition [73]. Nonetheless, according to the literature, it is both associated with undernutrition and diabetes [73–75].

In conclusion, while the quantitative analysis presented in this study provides a necessary foundation, it remains incomplete without a qualitative approach that captures the social and anthropological context, knowledge of which is essential for developing comprehensive and contextually appropriate public health policies. Qualitative or mixed-methods studies are needed to address the inherent limitations of quantitative analyses when examining epidemiologic and nutrition transitions. Accordingly, this analysis has been complemented by an ethnographic study of emic representations and hypotheses concerning the nutrition transition in the OPSE [76]. In addition, substantial efforts to improve data quality in under-resourced and remote settings are crucial for reducing the proportion of unknown and ill-defined information. Some of this work is already underway in Senegal’s OPSE, where demographic data collection methods have been harmonised and modernised, and the verbal autopsy questionnaire has been recently updated.

## Conclusion

Available mortality data from three rural areas of Senegal suggest that epidemiological trends align with the first phase of the epidemiologic transition (‘receding epidemics’ [3]) and an intermediate stage between the third and fourth patterns of the nutrition transition (‘receding famine’ and ‘degenerative diseases’ [5]). Specifically, the area under study appears to be undergoing a double burden, similar to other LMIC contexts: While communicable diseases and undernutrition are declining, they continue to contribute to mortality; and NCDs are emerging as a major health concern. The epidemiologic transition appears to be both initiated and plausibly accelerated by a nutrition transition. While the rise in NCDs is partly attributable to population-ageing, it is also likely that they are increasing among middle-aged adults due to emerging risk factors, including new forms of malnutrition. The growing impact of NCDs, even in rural areas, warrants further investigation and should be addressed in national and international public health strategies. Without such attention, gains in life expectancy achieved through progress against undernutrition, maternal mortality, and infectious diseases may be offset later in life by complex chronic conditions that remain poorly prevented and managed in rural settings. Mixed-methods research is especially needed to better understand how evolving environments and lifestyles in rural areas of low-income countries affect risk factors and challenge local health systems. Improving the availability and quality of health data in rural areas of LMICs should also be prioritised.

## Supplementary Material

Supplemental Material

## Data Availability

The data used for this article are held by OPSE and may be requested as part of a relevant research project.

## Abbreviations

LMICs: Low- and middle-income countries; WHO: World Health Organization; GBD: Global Burden of Disease; HDSS: Health and Demographic Surveillance System; NCD: non-communicable disease; CMM: Communicable diseases, Maternal disorders and Undernutrition; UNSD: United Nations Statistics Division.
