# Supplementary material for: Adult mortality and nutrition in rural Senegal: evidence of an epidemiologic transition
Source: Glob Health Action. 2025 Sep 12;18(1):2547493. doi: 10.1080/16549716.2025.2547493 (PMC12434866; doi:10.1080/16549716.2025.2547493)
Supplement: Supplemental Material [file ZGHA_A_2547493_SM1283.docx]

# Supplemental material

## Distribution of unknown and ill-defined causes

Unknown and ill-defined causes are unevenly distributed across age groups, sites, and time periods. [Figure S1](#fig-undefined) provides an overview of this distribution for each site and age group.

| 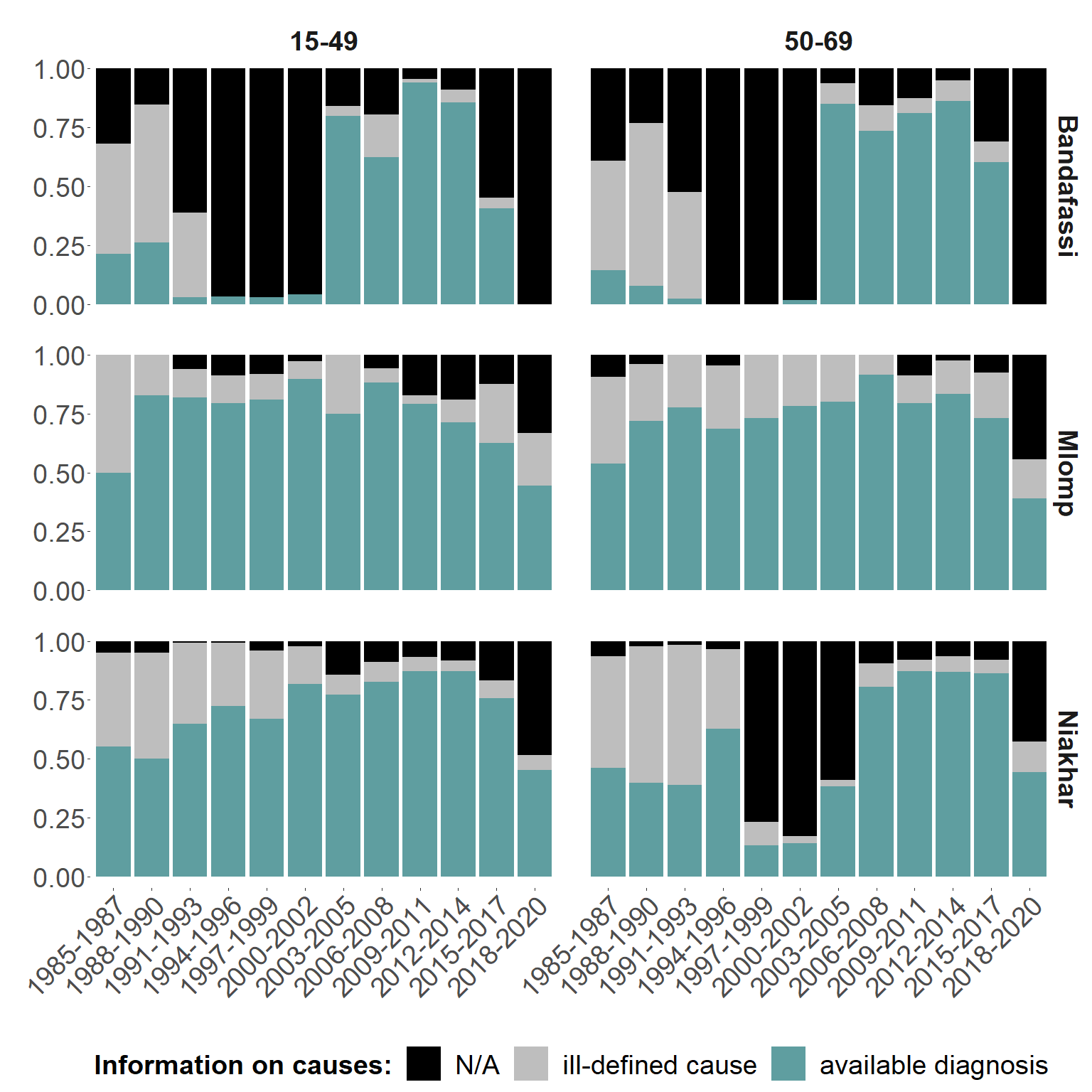  Figure S1: Availability of causes of death in Bandafassi, Mlomp and Niakhar (Senegal HDSS), 1985–2020, population aged 15–70.  Source: OPSE data (authors’ calculations) |
| --- |

## Description of the cause-specific analysis dataset

[Figure S2](#fig-dbcod) shows the distribution of the study population across sites by age and period groups.

| 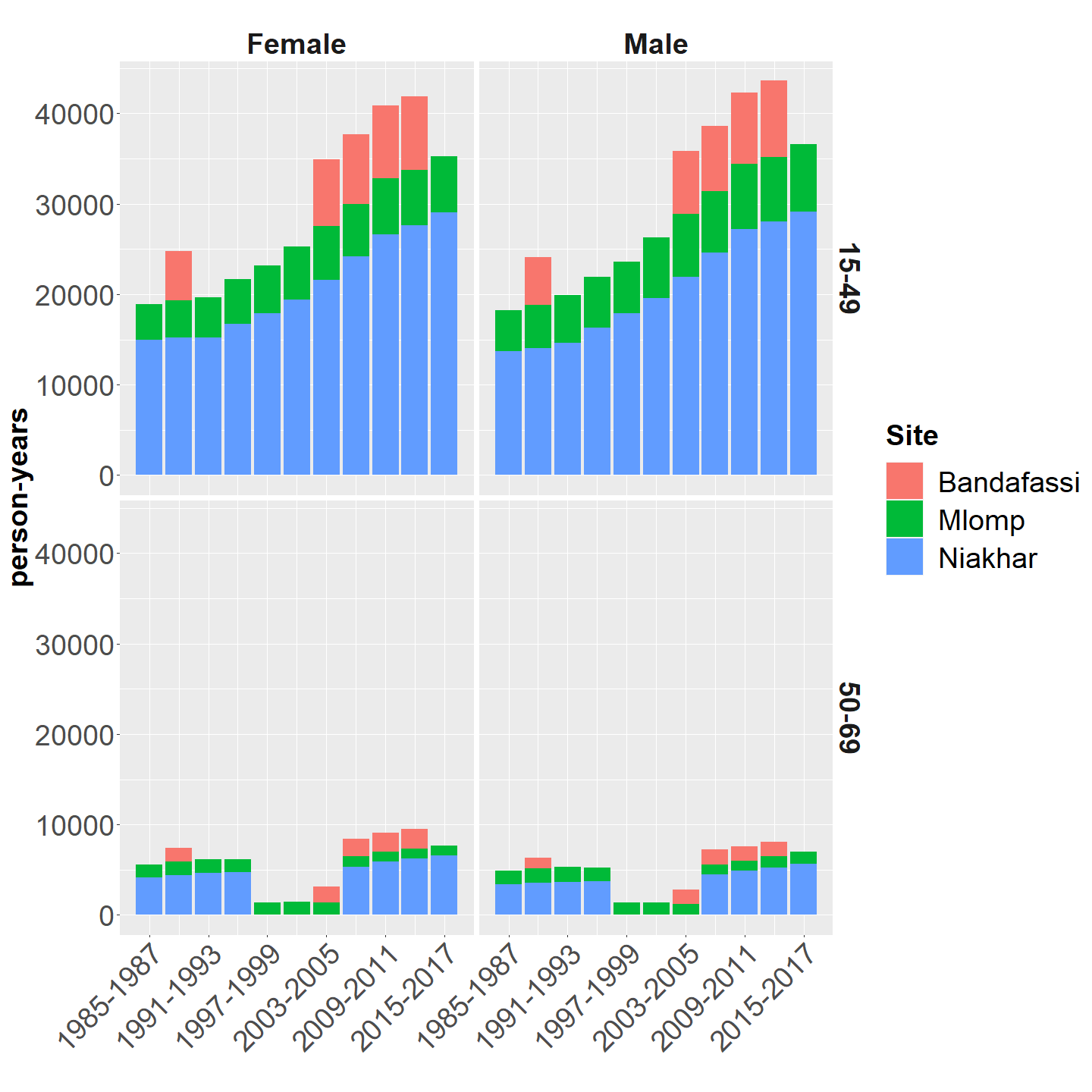  Figure S2: Detailed composition of the study population for cause-specific analyses (Senegal HDSS), 1985–2017, population aged 15–70.  Source: OPSE data (authors’ calculations) |
| --- |

[Table S1](#tbl-datacod) describes the dataset used for the cause-specific analysis.

Table S1: Description of the dataset for the cause-specific analysis (Senegal HDSS), 1985–2017, population aged 15–70.

|  | Bandafassi | Mlomp | Niakhar | Total |
| --- | --- | --- | --- | --- |
| Individuals 1985–2017 | 11164 | 13721 | 47107 | 71,992 |
| Person-years 1985–2017 | 89,724 | 156,477 | 531,916 | 778,117 |
| Deaths 1985-2017 | 637 | 845 | 2857 | 4339 |
| *> causes of death N/A* | 81 | 40 | 180 | 301 |
| *> ill-defined causes* | 122 | 162 | 690 | 974 |
| *> defined causes* | 434 | 684 | 1987 | 3105 |

*Source: OPSE data (authors’ calculations)*

## Nutrition-related death rates and ill-defined causes

[Figure S3](#fig-drnut_ill-defined) presents standardised death rates from nutrition-related causes alongside standardised death rates from ill-defined causes.

| 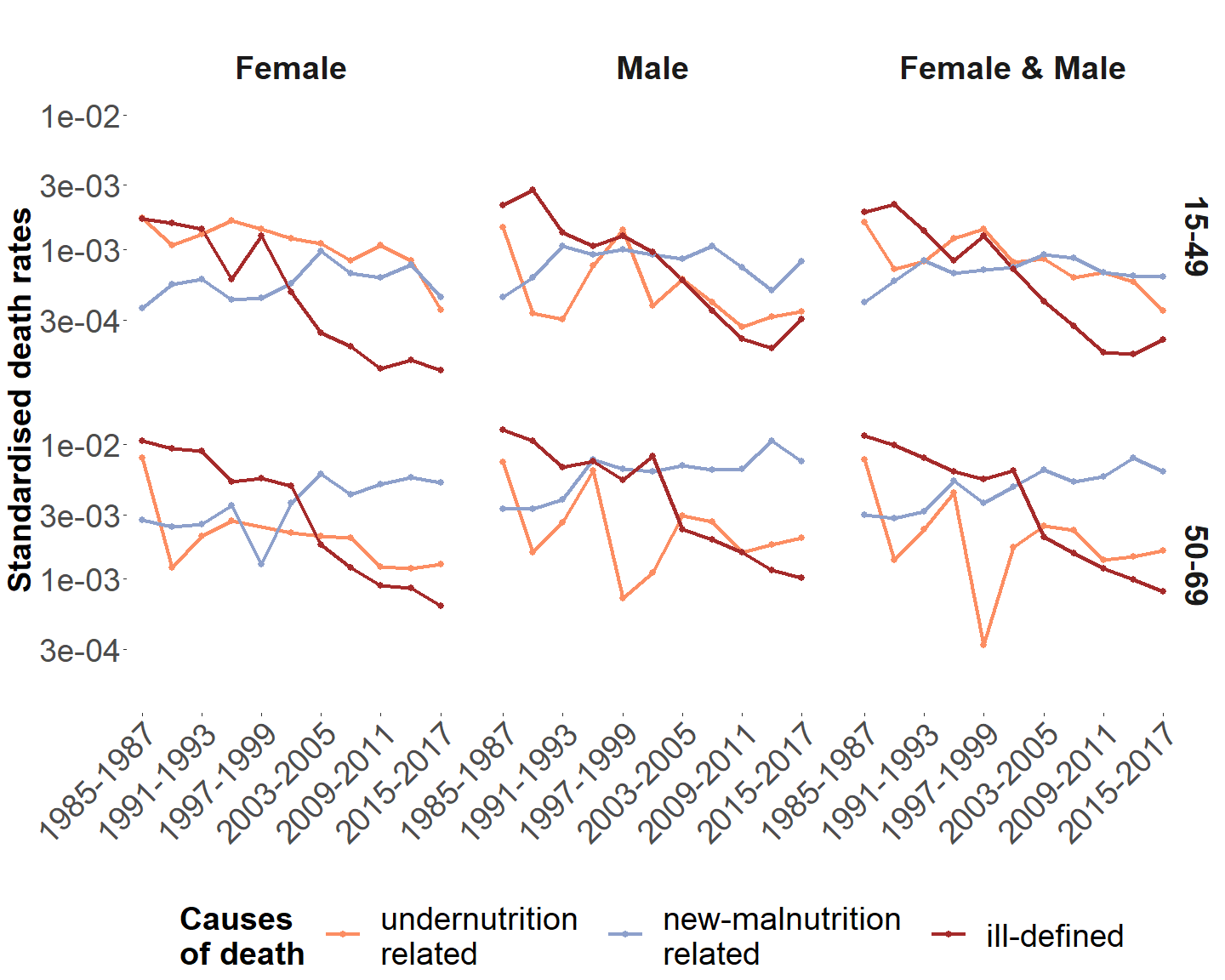  Figure S3: Standardised death rates for nutrition-related and ill-defined causes of death (Senegal HDSS), 1985–2017, population aged 15–70.  Source: OPSE data (authors’ calculations) |
| --- |
